# Drug resistance markers within an evolving efficacy of anti-malarial drugs in Cameroon: a systematic review and meta-analysis (1998–2020)

**DOI:** 10.1186/s12936-020-03543-8

**Published:** 2021-01-09

**Authors:** Peter Thelma Ngwa Niba, Akindeh M. Nji, Marie-Solange Evehe, Innocent M. Ali, Palmer Masumbe Netongo, Randolph Ngwafor, Marcel N. Moyeh, Lesley Ngum Ngum, Oliva Ebie Ndum, Fon Abongwa Acho, Cyrille Mbanwi Mbu’u, Dorothy A. Fosah, Barbara Atogho-Tiedeu, Olivia Achonduh-Atijegbe, Rosine Djokam-Dadjeu, Jean Paul Kengne Chedjou, Jude D. Bigoga, Carole Else Eboumbou Moukoko, Anthony Ajua, Eric Achidi, Esther Tallah, Rose G. F. Leke, Alexis Tourgordi, Pascal Ringwald, Michael Alifrangis, Wilfred F. Mbacham

**Affiliations:** 1 MARCAD-DELTAS Programme, Laboratory for Public Health Research Biotechnologies, University of Yaoundé I, Yaoundé, Cameroon; 2 The Biotechnology Centre, University of Yaoundé I, Yaoundé, Cameroon; 3 Department of Biochemistry, Faculty of Science, University of Yaoundé I, Yaoundé, Cameroon; 4 Department of Biochemistry, Faculty of Science, University of Dschang, Dschang, Cameroon; 5 National Malaria Control Programme, Ministry of Public Health, Yaoundé, Cameroon; 6 Department of Biochemistry and Molecular Biology, Faculty of Science, University of Buea, Buea, Cameroon; 7 Department of Biochemistry, Faculty of Medicine and Biomedical Sciences, University of Yaoundé I, Yaoundé, Cameroon; 8 Institute of Medical Research and Medicinal Plant Studies, Ministry of Scientific Research and Innovation, Yaoundé, Cameroon; 9 Université Des Montagnes, Banganté, West Region Cameroon; 10 Department of Microbiology, Faculty of Science, University of Yaoundé I, Yaoundé, Cameroon; 11 Faculty of Medicine and Pharmaceutical Sciences, University of Douala, Douala, Cameroon; 12 Malaria Research Service, Centre Pasteur Cameroon, Yaoundé, Cameroon; 13 Malaria Consortium-Cameroon Coalition Against Malaria, Yaoundé, Cameroon; 14 The Cameroon Office of the World Health Organization, Yaoundé, Cameroon; 15 Global Malaria Programme, World Health Organization, Geneva, Switzerland; 16 Centre for Medical Parasitology, Department of Immunology and Microbiology, Faculty of Health and Medical Sciences, University of Copenhagen, Copenhagen, Denmark; 17 Department of Infectious Diseases, Copenhagen University Hospital, Copenhagen, Denmark

**Keywords:** Malaria, *Plasmodium falciparum*, Anti-malarial drug, Resistance, Mutations, Efficacy, Systematic review, Cameroon

## Abstract

**Background:**

Malaria remains highly endemic in Cameroon. The rapid emergence and spread of drug resistance was responsible for the change from monotherapies to artemisinin-based combinations. This systematic review and meta-analysis aimed to determine the prevalence and distribution of *Plasmodium falciparum* drug resistance markers within an evolving efficacy of anti-malarial drugs in Cameroon from January 1998 to August 2020.

**Methods:**

The PRISMA-P and PRISMA statements were adopted in the inclusion of studies on single nucleotide polymorphisms (SNPs) of *P*. *falciparum* anti-malarial drug resistance genes (*Pfcrt*, *Pfmdr1*, *Pfdhfr*, *Pfdhps*, *Pfatp6*, *Pfcytb* and *Pfk13*). The heterogeneity of the included studies was evaluated using the Cochran’s Q and I^2^ statistics. The random effects model was used as standard in the determination of heterogeneity between studies.

**Results:**

Out of the 902 records screened, 48 studies were included in this aggregated meta-analysis of molecular data. A total of 18,706 SNPs of the anti-malarial drug resistance genes were genotyped from 47,382 samples which yielded a pooled prevalence of 35.4% (95% CI 29.1–42.3%). Between 1998 and 2020, there was significant decline (P < 0.0001 for all) in key mutants including *Pfcrt* 76 T (79.9%-43.0%), *Pfmdr1* 86Y (82.7%-30.5%), *Pfdhfr* 51I (72.2%-66.9%), *Pfdhfr* 59R (76.5%-67.8%), *Pfdhfr* 108 N (80.8%-67.6%). The only exception was *Pfdhps* 437G which increased over time (30.4%-46.9%, P < 0.0001) and *Pfdhps* 540E that remained largely unchanged (0.0%-0.4%, P = 0.201). Exploring mutant haplotypes, the study observed a significant increase in the prevalence of *Pfcrt* CVIET mixed quintuple haplotype from 57.1% in 1998 to 57.9% in 2020 (P < 0.0001). In addition, within the same study period, there was no significant change in the triple *Pfdhfr* IRN mutant haplotype (66.2% to 67.3%, P = 0.427). The *Pfk13* amino acid polymorphisms associated with artemisinin resistance were not detected.

**Conclusions:**

This review reported an overall decline in the prevalence of *P. falciparum* gene mutations conferring resistance to 4-aminoquinolines and amino alcohols for a period over two decades. Resistance to artemisinins measured by the presence of SNPs in the *Pfk13* gene does not seem to be a problem in Cameroon.

*Systematic review registration* PROSPERO CRD42020162620

## Background

Globally, malaria accounted for 228 million cases and 405,000 related deaths in 2018 [1]. Malaria remains highly endemic in Cameroon despite the adoption, implementation and deployment of different controls measures by the government and her partners [1]. In Cameroon, the rapid emergence and spread of anti-malarial drug resistance was responsible for the replacement of chloroquine (CQ) as the first-line therapy for treatment of uncomplicated *Plasmodium falciparum* malaria in 2002 and later on amodiaquine (AQ) monotherapy/sulfadoxine-pyrimethamine between 2002 and 2004 [2]. A major drug policy change occurred in 2004 following the adoption of artesunate-amodiaquine (ASAQ) and later included artemether–lumefantrine (AL) in 2006 as first-line treatments of uncomplicated malaria in line with World Health Organization (WHO) recommendations [2, 3]. The artemisinin-based combinations, ASAQ and AL, are distributed in the proportions of 75% and 25%, respectively to public, faith-based and private health facilities [4]. In the Northern Regions of Cameroon, malaria transmission is intense and seasonal when compared to the Southern Regions characterized with perennial malaria transmission. In 2016, the government of Cameroon implemented seasonal malaria chemoprevention (SMC) in the Northern Regions [5]. This prevention strategy involves the yearly administration of four doses of sulfadoxine–pyrimethamine–amodiaquine (SPAQ) to vulnerable children within the age group 3–59 months [5]. Additionally, SP is still being used as an intermittent preventive treatment in pregnant (IPTp) women from the second to the third trimester. The women receive at least 3 doses during pregnancy, with each dose (three tablets of 500 mg sulfadoxine and 25 mg pyrimethamine) given at least 1 month apart [6, 7]. Both SPAQ and SP are also subsidized by the government of Cameroon. The large-scale deployment of the SMC and IPTp strategies is a major contributory factor to drug pressure which drives the emergence of *P. falciparum* resistant parasites.

Furthermore, the efficacy of anti-malarial drugs is linked to the presence or absence of parasites resistant to artemisinin-based combination therapy (ACT) and non-ACT in the population. Thus, the regular monitoring of drug resistance markers through molecular surveillance or clinical trials can be used by malaria control programmes in endemic regions to secure the high efficacy of the different anti-malarial drugs. The use of advanced molecular biology techniques has greatly facilitated the identification of key amino acid changes in the genes of *P*. *falciparum* chloroquine resistant transporter*-Pfcrt* (C72S, V73K, M74I, N75E, K76T, A220S, Q271E, N326S, I356T, R371I) [8], *P*. *falciparum* multi-drug resistant 1*-Pfmdr1* (N86Y, Y184F, S1034C, N1042D, D1246Y, copy number variation) [8], *P*. *falciparum* dihydrofolate reductase*-Pfdhfr* (A16V, C50R, N51I, C59R, S108N/T) [9–11] and *P*. *falciparum* dihydropteroate synthase*-Pfdhps* (I431V, S436A/F, A437G, K540E/N, A581G, A613S/T) [12] associated with resistance to different anti-malarial drugs. The presence of *Pfcrt* K76T is associated with increased risk of treatment failure after administration of chloroquine whereas, *Pfmdr1* N86Y is associated with both chloroquine and amodiaquine resistance [13]. The haplotypes of the *Pfcrt* gene defined by the K76T codon and adjacent amino acids (numbers 72–75) have been used in the typing of malaria parasites [14]. Among the over fifteen haplotypes identified, three predominate namely: CVMNK among CQ-sensitive isolates from all geographic regions, CVIET among CQ-resistant isolates from Southeast Asia and Africa, and **S**VMNT among CQ-resistant isolates from South America, Africa and some countries of Asia [14–16]. For sulfadoxine–pyrimethamine the *Pfdhfr* single (S108N), triple haplotype mutants (S108N, N51I, C59R) and *Pfdhfr-Pfdhps* quintuple haplotype mutants (S108N, N51I, C59R, A437G, K540E) have been shown to increase the risk of treatment failure [13]. It has also been documented that increased *Pfmdr1* copy number is correlated with resistance to mefloquine [17] and reduced sensitivity to lumefantrine [18–20]. A study on AL and ASAQ showed opposing effects for *Pfcrt* K76T and *Pfmdr1* N86Y [21]. This was further confirmed by another study on the selection of *Pfmdr1* NFD haplotype for AL and *Pfmdr1* YYY haplotype for ASAQ from samples of efficacy studies conducted in Africa that led to reduced sensitivities of the two drugs [22].

In 2014, single nucleotide polymorphisms in the *Pfk13* propeller domain of Cambodian parasite isolates were reported to be associated with delayed parasite clearance of artemisinins [23]. The epicentres driving the emergence and dispersal of artemisinin resistance have been identified in countries within the Greater Mekong sub-region (GMS) namely, Cambodia, China (Yunnan Province), Lao People’s Democratic Republic, Myanmar, Thailand and Vietnam [24]. Presently, about 200 non-synonymous mutations in the K13 gene have been identified and reported [24–27]. A total of 9 *Pfk13* non-synonymous single nucleotide polymorphisms (F446I, N458Y, N458Y, Y493H, R539T, I543T, P553L, R561H, C580Y) have been validated with F446I, R539T, I543T, P574L and C580Y being the most common and with the highest occurrences [24, 25, 27]. There are 11 candidate gene polymorphisms associated with delayed parasite clearance [24, 25, 27]. A number of mutations have also been reported outside the K13 propeller region notably, K189T and E252Q [25, 28–30]. In Africa, the *Pfk13* mutation with the highest geographical distribution is A578S [25, 26, 31] and the presence of R561H mutation has recently been reported in Tanzania [32] and Rwanda [33]. Hence, there are fears that ACT resistance may spread to other regions including sub-Saharan Africa where malaria is still a major burden, similar to what happened in the past with the chloroquine, amodiaquine, and sulfadoxine–pyrimethamine. The rationale for the use of ACT relies on the rapid reduction of the parasite biomass, reduction of transmission (reducing gametocytes), protection of partner drug against resistance, and rapid fever reduction [34]. The effect of drug policy changes on the selection of *P*. *falciparum* anti-malarial drug resistant parasites in Cameroon has not been completely understood. Therefore, this systematic review and meta-analysis aimed to determine the prevalence and distribution of *P*. *falciparum* drug resistance markers within an evolving efficacy of anti-malarial drugs in Cameroon from January 1998 to August 2020.

## Methods

### Registration of the systematic review and protocol development

In December 2019, a review protocol (#CRD42020162620) was developed and registered in the International Prospective Register of Systematic Reviews (PROSPERO: http://www.crd.york.ac.uk/prospero). The protocol was submitted for publication to a peer review journal. The Preferred Reporting Items for Systematic Reviews and Meta-analyses Protocol (PRISMA-P) [35, 36] was used in the development of the protocol for this systematic review and meta-analysis.

### Search strategy

An electronic systematic strategy based on the combination of key words was used to search articles from Medline via Pubmed, Google Scholar, and Science Direct databases. Both interventional and observational studies were retrieved for inclusion in the review. The following MeSH search terms were combined using the Boolean operators “OR” and “AND’’: “anti-malarial”, “drug resistance”, “*Pfcrt*”, “*Pfmdr1*”, “*Pfmdr1 copy number*”, “*Pfdhfr*”, “*Pfdhps*”, “*Pfatp6*”, “*Pfcytb*”, “*Pfk13*”, “mutations”, “gene polymorphisms”, “amino acid changes”, “*Plasmodium falciparum*”, “efficacy”, “artesunate-amodiaquine”, “artemether–lumefantrine”, “sulfadoxine–pyrimethamine” “Cameroon”.

### Additional searches

The reference lists of published articles were searched for eligible studies. Authors were contacted when access to full length articles was restricted. Data was also obtained from the annual reports of the Cameroon National Malaria Control Programme (NMCP), Ministry of Public Health. In addition to published studies, unpublished Medical Doctor (MD), Master of Science (MSc) and Doctor of Philosophy (PhD) theses were sourced for inclusion in the study.

### Eligibility criteria

#### Inclusion criteria

The systematic review and meta-analysis included the following type of studies: studies published from January 1998 to August 2020; studies on human participants of all ages; original articles of studies that investigated either asymptomatic, uncomplicated or severe *P*. *falciparum*; studies that included PCR genotyping of anti-malarial drug molecular resistance markers (*Pfcrt*, *Pfmdr1*, *Pfmdr1* copy number, *Pfdhfr*, *Pfdhps*, *Pfcytb*, *Pfatp6, Pfk13*); studies written in English or French; studies done within Cameroon: all multi-centric studies in which Cameroon was one of the sites, and studies in which malaria was imported from Cameroon into other countries.

#### Exclusion criteria

The following types of studies were not included: abstracts; studies on in vitro, ex vivo and in vivo anti-malarial drug resistance without genotyping; genetic studies on *Pfcg2* gene; studies on genetic diversity and population structure of *P. falciparum* without drug resistance; studies on diagnostic accuracy of methods for detection of *P*. *falciparum* and studies on infections with mixed *Plasmodium* species.

#### Review process

Research articles identified from searches of the electronic databases were screened for eligibility based on their titles and abstracts. Ineligible articles and duplicates were eventually removed. Full-length articles of the selected studies were read to confirm for fulfilling of the inclusion criteria before data extraction began. Two independent reviewers (Peter Thelma Ngwa Niba-PTNN and Lesley Ngum Ngum-LNN) screened the titles and abstracts to identify potentially eligible studies and data extracted from full-length articles that fulfilled the inclusion criteria. Discrepancies were resolved by mutual consent after discussion and independent review from the third researcher (Akindeh Mbuh Nji-AMN). The whole process was supervised by Wilfred Fon Mbacham (WFM) and Michael Alifrangis (MA).

### Data extraction procedure

The “Microsoft” Excel 2010 (Microsoft Corporation, Redmond, Washington, United States of America) was used to design the data extraction sheet. The data extraction form was produced and consisted of study identification number, author (s), study site, sample size, age group (in months and years), study design (interventional and observational), genotyping method, sequence genotyping success rate, anti-malarial drug resistance gene, total number of samples genotyped, number of samples genotyped with mutations, and prevalence of molecular markers. The database in Microsoft Excel was piloted and validated before completion of the process (Additional file 1).

Mixed genotypes were considered as mutants during data collation on frequency of mutations derived from different studies. Studies (observational or interventional) published multiple times in similar topics by the same authors were diligently screened to avoid duplication of data. These studies were differentiated based on primary variables (anti-malarial drug resistance markers and frequency of single nucleotide polymorphisms) containing the datasets of interest. The PRISMA (Preferred Reporting Items for Systematic Reviews and Meta-Analyses) checklist for reporting systematic reviews and meta-analyses was used as a guide for this study [37].

### Data items

The selection and inclusion of studies was done according to the PICOS format. This approach includes: population (P), individuals infected *P*. *falciparum* parasites in Cameroon, intervention (I), use of non-artemisinin and artemisinin agents in the treatment of malaria, comparator (C), none, outcome (O), *Pfcrt*, *Pfmdr1*, *Pfdhfr*, *Pfdhps*, *Pfk13* gene polymorphisms circulating in malaria endemic areas of Cameroon, study design (S), observational studies (cross-sectional, case reports, cohorts) and interventional studies such as randomized controlled trials reporting on the use of *P*. *falciparum* DNA infected samples collected before anti-malarial treatment (D0) and during follow-ups of study participants.

### Data management

The Zotero Standalone software package version 5.0.56 (Corporation for Digital Scholarship, Vienna, Virginia, USA) was used to review, import full articles and delete duplicates.

### Methodological quality (risk of bias) assessment of individual studies included

The quality of randomized clinical trials was assessed by the revised Cochrane risk of bias tool for randomized trials (RoB 2.0) [38]. The RoB 2 is structured into five bias domains namely: bias arising from the randomization process, bias due to deviations from intended interventions, bias due to missing outcome data, bias in measurement of the outcome, and bias in selection of the reported result. The overall quality of the randomized clinical trial was judged as “low risk” of bias score when all the key domains in the assessment of bias were found to be of low risk. When one of the key domains in the bias assessment was found to have some concerns, a scoring of “some concerns” was rendered. The assessment of at least one key domain of bias with a high risk in a study accorded it to be of “high risk” of bias (Additional file 2). The quality of cohort studies was assessed using the Newcastle–Ottawa Scale (NOS), which included eight items related to selection, comparison, and outcome. For each item a star is awarded except for comparison that can receive up to two stars. The studies with six stars (maximum of nine) were classified as good quality (Additional file 3) [39]. Finally, the quality of included cross-sectional studies and case reports was assessed by the Joanna Briggs Institute (JBI) Critical Appraisal Checklists for Cross-sectional [40] and Case Reports [41] which consist of eight yes/no/unclear questions. The overall quality of cross-sectional and case reports were grouped into the following categories: low risk of bias (studies that met at least 75% of the quality criteria), moderate risk of bias (studies that met between 50 and 74% of the quality criteria) and high risk of bias (studies that met less than 49% of the quality criteria) (Additional files 4 and 5) [42].

Two reviewers (Peter Thelma Ngwa Niba-PTNN and Cyrille Mbanwi Mbu'u-CMM) independently assessed the risk of bias of included studies. Disagreements between the reviewers at the different stages of the review were resolved by discussion.

### Data analysis, heterogeneity assessment and data interpretation

Quantitative syntheses (meta-analyses) were done using the “metaphor” and “meta” packages in the R statistical software version 3.5.2 (supported by the R Foundation for Statistical Computing, Vienna, Austria). The conventional meta-analysis approach from pooled patient data was adopted for the synthesis. The heterogeneity of the included studies was evaluated using the Cochran’s Q and I^2^ statistics. The random effects model was used as standard in the determination of heterogeneity between studies [43]. The I^2^ values were expressed in percentages. Heterogeneity was classified as low, moderate and high, with upper limits of 25%, 50% and 75% for I^2^, respectively [44].

Data derived from an article published by one author or same authors in a particular year were merged before presentation on forest plots. Forest plots were used to present the data on pooled prevalence of mutations in anti-malarial drug resistance genes. Subgroup analyses were also done to show the aggregated prevalence of *Pfcrt* K76T, *Pfmdr1* N86Y, *Pfdhfr* IRN haplotype, *Pfdhfr-Pfdhps* IRNG haplotype and *Pfk13* gene mutations in cases where number of studies were greater than or equal to 5. The evolution of resistance markers and haplotypes over time was summarized on frequency tables.

The pre and post-ACT intervention periods were considered to be 1998–2004 and 2005–2020 respectively. The criterion for choosing these periods was based on 2004, the year in which the first ACT was adopted for use in Cameroon. In the analysis to compare SNPs between the two or more study periods, mixed infections with both the wild type and the mutant were all considered mutants. Haplotypes were defined as a combination of two or more wild type alleles, mutant alleles or mixed. These haplotypes included *Pfcrt* CVMNK, *Pfcrt* CVIET, *Pfdhfr* IRN, *Pfdhfr-Pfdhps* IRNG, and *Pfdhfr-Pfdhps* IRNGE.

The Pearson Chi square test in the International Business Machine Software Package for Social Sciences (IBM SPSS) version 20.0 software package (IBM Corporation, Armonk, New York, USA) was used to establish the evolution of drug resistance markers over time.

The Shapiro–Wilk test was used to check for normal distribution of quantitative variable data. Furthermore, the relationships between the efficacy of ACT medicines (ASAQ and AL) and anti-malarial drug resistance makers (*Pfcrt* 76 T and *Pfmdr1* 86Y) were represented on plots. The Pearson Correlation Coefficient (r) was used to assess the strength and direction of the association between the efficacy of ACT medicines (AL and ASAQ) and the prevalence of *Pfcrt* 76 T and *Pfmdr1* 86Y mutants over time. In addition, a trend analysis to explore the relationship between proportions of anti-malarial drugs (ASAQ, AL and SP) deployed in Cameroon and prevalence of drug resistance markers (*Pfcrt* 76 T, *Pfmdr1* 86Y and *Pfdhfr* IRN) from 2006 to 2017 was also explored using r. The standard range for r values is between -1 and + 1. The level of significance was set at p < 0.05 at 95% confidence interval and two-tailed.

### Assessment of publication bias across studies

The risk of publication bias in the included articles was assessed using the asymmetry of funnel plot and Egger’s regression test with P < 0.05. The funnel plot contained the standard error on the y-axis and proportion on the x-axis (Additional file 6).

## Results

### Study identification, screening and selection process

**Fig. 1:**
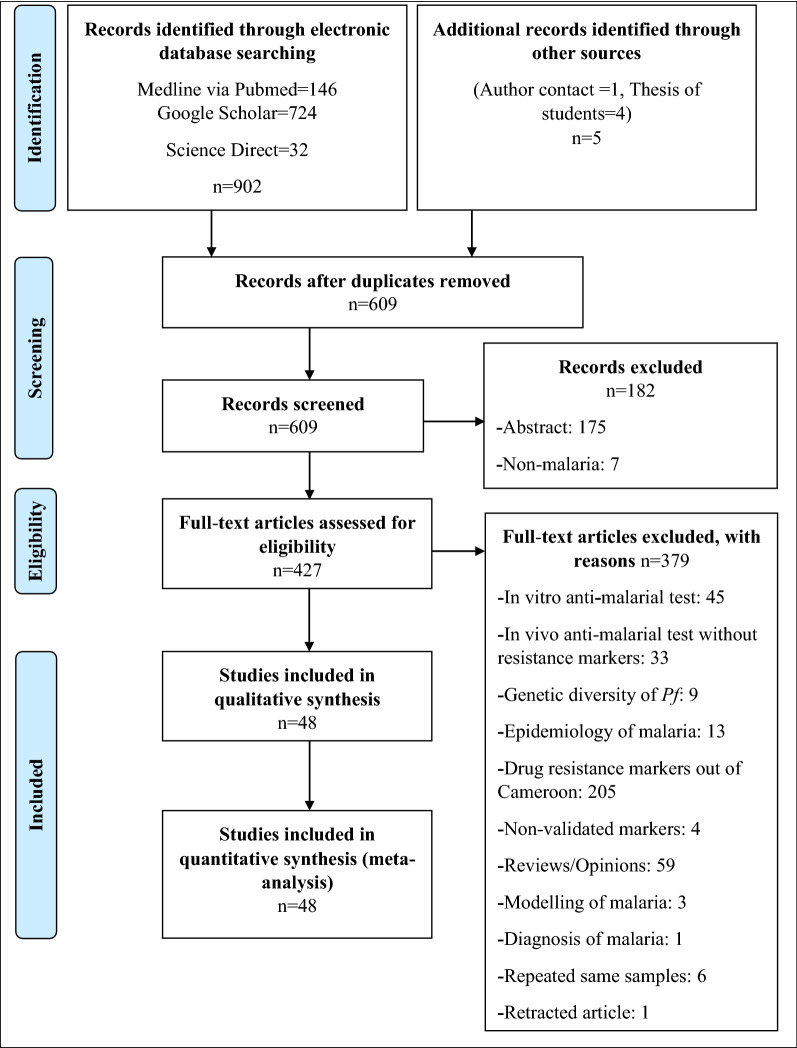
Flow chart for studies included in the systematic review and meta-analysis on anti-malarial drug resistance markers in Cameroon from 1998–2020

The electronic searches identified a total of 902 published articles on anti-malarial drug resistance markers in Cameroon. There were three additional unpublished citations from theses of students and one article was derived through contact with a senior researcher on malaria disease. A total of 907 studies were identified, after which 298 duplicates were removed. A total of 609 studies were screened to remove abstract and non-malaria studies, with 427 studies retained after the process. The 427 studies were checked for eligibility, with 48 studies included for both qualitative and quantitative analyses (Fig. 1).

### Characteristics of studies included in the review

Participants of all age groups ranging from 0 months to 80 years and both gender were included in the study. Out of 48 studies included, 44 studies [26, 28, 30, 45–85] were obtained from published articles and 4 from unpublished data. A total of 38 (79.2%) were carried out only in Cameroon, 7 (14.6%) were studies of imported malaria cases from African countries including Cameroon, and 3 (6.3%) were multi-centric studies including Cameroon. The studies were performed in all the geo-ecological zones constituted from the 10 Regions of Cameroon, that is, Sudano-Sahelian, tropical, and equatorial. Majority of these studies (n = 25 (52.1%)) were conducted in Yaoundé. Most of the studies (n = 25 (52.1%)) were derived from observational studies while the remaining studies were randomized controlled clinical trials. The main methods used for genotyping were nested polymerase chain reaction-restriction fragment length polymorphism (nPCR-RFLP), DNA sequencing by Sanger (sequencing by dideoxy-chain termination method) and quantitative real time polymerase chain reaction. Others methods included sequence specific oligonucleotide probe, polymerase chain reaction, enzyme linked immunosorbent assay (SSOP PCR ELISA), dot blot, and DNA sequencing using Illumina HiSeq platform. A total of seven *P*. *falciparum* drug resistance genes datasets were created for quantitative syntheses with the following distribution of studies: *Pfcrt* (n = 17), *Pfmdr1* (n = 15), *Pfdhfr* (n = 21), *Pfdhps* (n = 13), *Pfcytb* (n = 1), *Pfatp6* (n = 2) and *Pf13* (n = 7) (Additional file 1).

### Heterogeneity of included studies

The assessment of heterogeneity was done for all the groups containing different studies on *P*. *falciparum* single nucleotide polymorphisms that confer resistance to anti-malarial drugs. There was high heterogeneity across all the groups: pooled prevalence of all drug resistance markers (Q(df = 41) = 43,100.1, I^2^ = 99%, P < 0.0001), *Pfcrt* K76T (Q(df = 20) = 999.5, I^2^ = 96%, P < 0.0001), *Pfcrt* CVIET (Q(df = 8) = 1588.9, I^2^ = 94%, P < 0.0001), *Pfmdr1* N86Y (Q(df = 15) = 1745.4, I^2^ = 97%, P < 0.0001), *Pfdhfr* IRN (Q(df = 25) = 4943.6, I^2^ = 96%, P < 0.0001), *Pfdhfr-Pfdhps* IRNG (Q(df = 10) = 64.4, I^2^ = 84%, P < 0.0001), and *Pfk13* (Q(df = 6) = 72.1, I^2^ = 90%, P < 0.0001).

### Pooled prevalence of P. falciparum anti-malarial drug resistance mutations

**Fig. 2:**
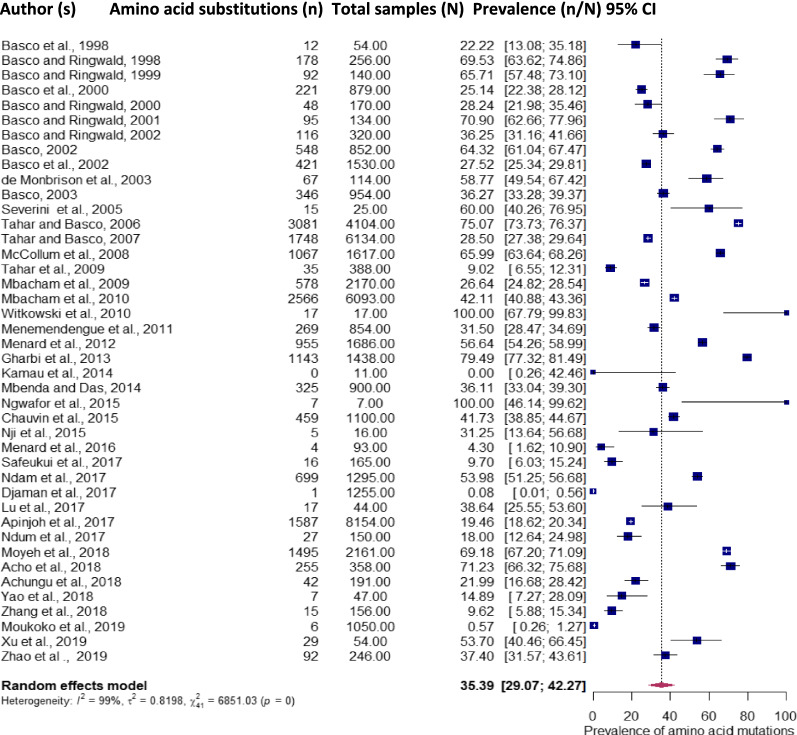
Pooled prevalence of *Plasmodium falciparum* anti-malarial drug resistance mutations from 1998–2020

There were 18,706 SNPs of anti-malarial drug resistance markers genotyped from 47,382 samples which yielded a pooled prevalence of 35.4% (95% CI 29.1–42.3%). The DNA sequence genotyping success rate varied from 45.1% to 100.0% while the prevalence of mutations ranged from 0.0 to 100.0% (Additional file 1 and Fig. 2).

The key amino acid substitutions represented in the analyses were: *Pfcrt* (C72S, V73K, M74I, N75E, K76T, A220S, Q271E, N326S, I356T, R371I), *Pfmdr1* (N86Y, Y184F, S1034C, N1042D, D1246Y, copy number variation), *Pfdhfr* (A16V, C50R, N51I, C59R, S108N/T) and *Pfdhps* (I431V, S436A/F, A437G, K540E, A581G, A613S/T). Only two studies recorded the presence of *Pfdhps* I431V with prevalence rates of 16.3% and 18.3% [30, 60]. One study reported the presence of *Pfdhps* K142N mutation with a prevalence of 8.5% not previously documented in Cameroon [30]. For *Pfk13*, the amino acid polymorphisms associated with artemisinin resistance in Southeast Asia were not detected in any of the 5912 *P*. *falciparum* samples genotyped and most of *Pfk13* gene polymorphisms reported here have not been observed anywhere in the world. The most prevalent non-validated *Pfk13* missense polymorphisms were K189T reported in 2 studies with prevalence rates of 21.2% and 35.9% [28, 30] (Additional file 1).

**Fig. 3:**
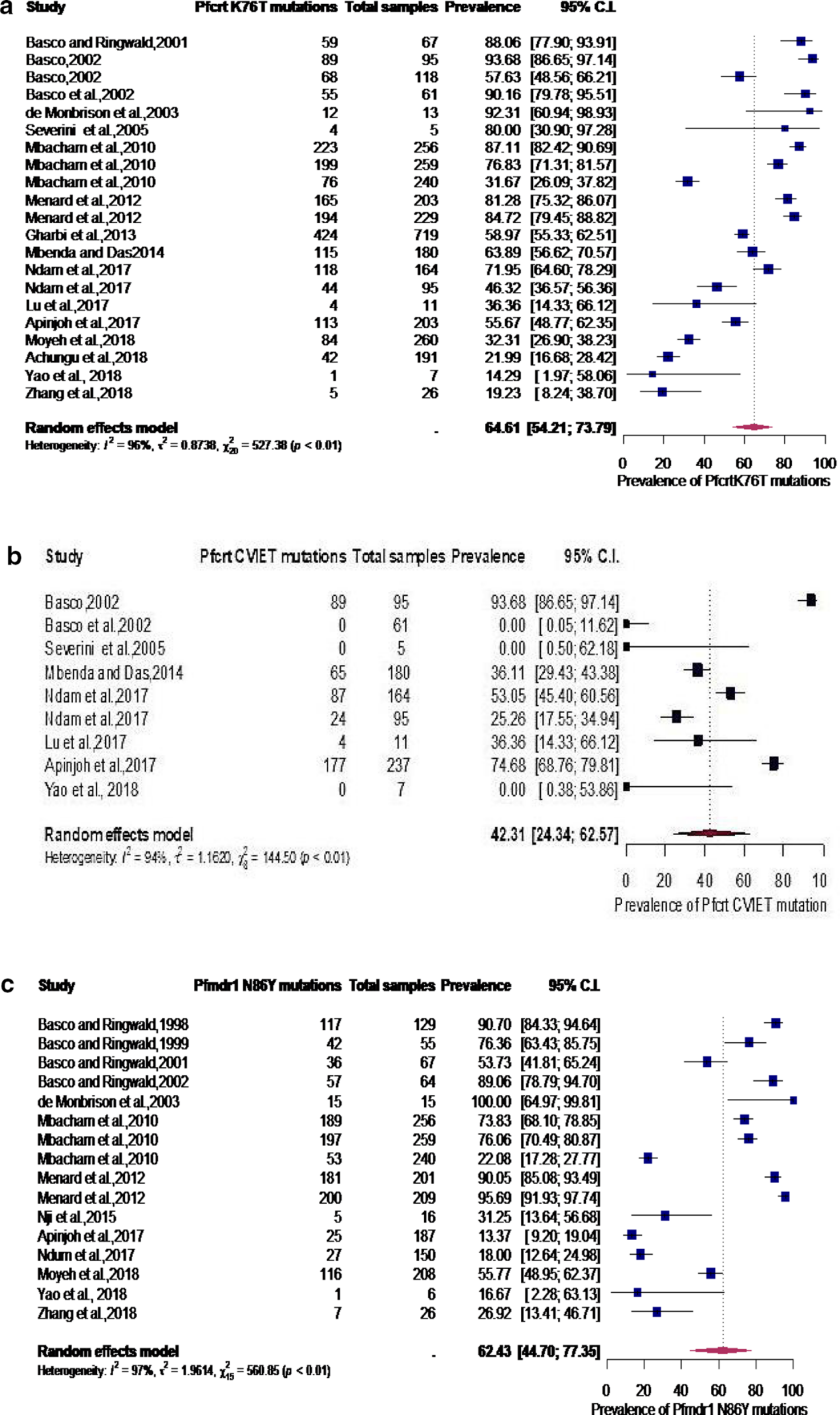

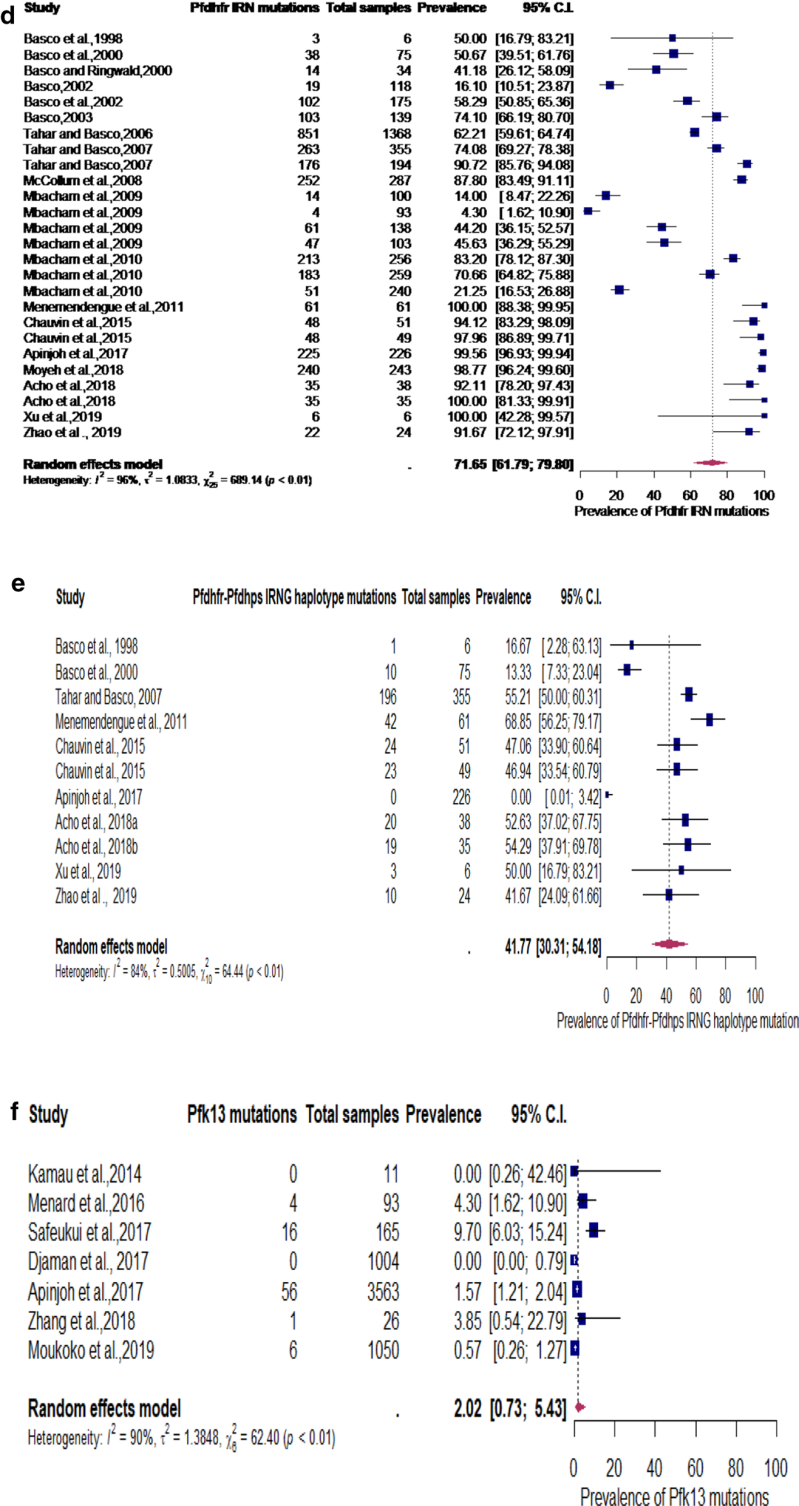
**a** Subgroup analysis for pooled prevalence of *Pfcrt* K76T mutation from 1998 to 2020. **b** Subgroup analysis for pooled prevalence of *Pfcrt* CVIET haplotype mutations from 1998–2020. **c** Subgroup analysis for pooled prevalence of *Pfmdr1* N86Y haplotype mutations from 1998 to 2020. **d** Subgroup analysis for pooled prevalence of *Pfdhfr* IRN haplotype mutations from 1998 to 2020. **e** Subgroup analysis for pooled prevalence of *Pfdhfr*-*Pfdhps* IRNG haplotype mutations from 1998 to 2020. **f** Subgroup analysis for pooled prevalence of *Pfk13* mutations from 1998 to 2020

Subgroup analyses revealed that the aggregated prevalence of *Pfcrt* K76T, *Pfcrt* CVIET, *Pfmdr1* N86Y, *Pfdhfr* IRN, and *Pfk13* genes were 64.6% (2094/3402) [95% CI 54.2–73.8%], 42.3% (446/855) [95% CI 24.3–62.6%], 62.4% (1268/2088) [95% CI 44.7–77.4%], 71.7% (3114/4673) [95% CI 61.8–79.8%], 41.8% (348/926) [95% CI 30.3–54.2%] and 2.0% (83/5912) [95% CI 0.7–5.4%] respectively (Fig. 3a–f).

### Temporal changes of the key gene polymorphisms conferring resistance to anti-malarial drugs prior to and after adoption of artemisinin-based combination therapies (ACTs) in Cameroon

The pre-ACT and post-ACT interventions were considered as periods before and after 2004 respectively. There was a significant decline in *Pfcrt* 76 T mutant alleles from 79.9% in 1998–2004 to 43.0% and 62.1% in 2005–2010 and ≥ 2017, respectively, with a slight increase of 67.5% recorded between 2011 and 2016 (P < 0.0001). Similarly, during the same study periods, the prevalence of the *Pfmdr1* 86Y mutant allele decreased significantly from 82.7% to 30.5% (P < 0.0001) with the exception observed between 2011 and 2016 when 90.6% was reported (Table 1).

**Table 1:** Changes in the frequency of *Pfcrt* and *Pfmdr1* genotypes between 1998 and 2020

Gene	Mutation	Allele	1998–2004 (%, n/N)	2005–2010 (%, n/N)	2011–2016 (%, n/N)	≥ 2017 (%, n/N)	P-value
*Pfcrt*	K76T	K76	20.1 (72/359)	37.9 (288/760)	32.5 (433/1331)	57.0 (546/958)	P < 0.0001*
		76 T	79.9 (287/359)	62.1 (472/760)	67.5 (898/1331)	43.0 (412/958)	
*Pfmdr1*	N86Y	N86	17.3 (57/330)	41.9 (317/756)	9.4 (40/426)	69.5 (401/577)	P < 0.0001*
		86Y	82.7 (273/330)	58.1 (439/756)	90.6 (386/426)	30.5 (176/577)	

*P < 0.05: Statistically significant, N: number of amino acid substitutions, N: Total number of samples genotyped

The only *Pfcrt* haplotypes reported in Cameroon were: CVMNK in 10 studies, CVIET in 6 studies and SVMNT in one study. The highest frequencies recorded were: CVMNK-85.7% [66], CVIET-93.7% [48], and SVMNT-4.4% [59] (Additional file 3). There was an increase in the prevalence of the *Pfcrt* CVMNK wild type haplotype from 7.7% in 1998–2004 to 20.0% in 2005–2010. However, a decrease of 15.9% was observed between 2010 and 2020. Generally, there was a significant increase in the prevalence rate of the CVMNK haplotype from 7.7% in 1998 to 40.2% in 2020 (P < 0.0001). The CVIET mutant haplotype declined from 57.1% in 1998–2004 to 0.0% in 2005–2010. The prevalence rate increased to 36.7% and 57.9% respectively in 2011–2016 and 2017–2020. Similarly, there was a significant increase in the prevalent rate of *Pfcrt* CVIET haplotype between 1998 and 2020 (P < 0.0001) (Table 2).

**Table 2:** Changes in the frequency of *Pfcrt* haplotypes between 1998 and 2020

Gene	Haplotype	1998–**2004** (%, n/N)	2005–**2010** (%, n/N)	2011–**2016** (%, n/N)	≥ 2017 (%, n/N)	P-value
*Pfcrt*	CVMNK	7.7 (12/156)	20.0 (1/5)	56.1 (101/180)	40.2 (323/804)	*P < 0.0001*
*Pfcrt*	CVIET	57.1 (89/156)	0.0 (0/5)	36.7 (66/180)	57.9 (301/520)	*P < 0.0001*

*P < 0.05: Statistically significant, n: Number of amino acid substitutions, N: Total number of samples genotyped

Within the *Pfmdr1* gene only the triple NFD haplotype was reported in 2 studies conducted in Mutengene [45, 57] with prevalence rates of 25.2% and 72.2%. The YFY and YYY triple haplotypes were not reported in any of the studies included (Additional file 3). Between the two time points 1998–2008 and 2009–2020, there was a significant drop (P < 0.0001) in the *Pfdhfr* (51I 72.2–66.9%, 59R 76.5–67.8%, 108 N 80.8–67.6%) mutant alleles whereas, the *Pfdhps* (437G 30.4–46.9%, P < 0.0001, 540E 0.0–0.4%, P = 0.201) mutant alleles increased over the two time points (Table 3).

**Table 3:** Changes in the frequency of *Pfdhfr* and *Pfdhps* genotypes between 1998 and 2020

Gene	Mutation	Allele	1998–2008 (%, n/N)	2009–2020 (%, n/N)	P-value
*Pfdhfr*	N51I	N51	27.8 (765/2751)	33.1 (624/1887)	P < 0.0001*
		51I	72.2 (1986/2751)	66.9 (1263/1887)	
	C59R	C59	23.5 (647/2751)	32.2 (621/1929)	P < 0.0001*
		59R	76.5 (2104//2751)	67.8 (1308/1929)	
	S108N	S108	19.2 (569/2959)	32.4 (622/1921)	P < 0.0001*
		108 N	80.8 (2390/2959)	67.6 (1299/1921)	
*Pfdhps*	A437G	A437	69.6 (477/685)	53.1 (1004/1890)	P < 0.0001*
		437G	30.4 (208/685)	46.9 (886/1890)	
	K540E	K540	100.0 (433/433)	99.6 (1849/1856)	P = 0.201
		540E	0.0 (0/433)	0.4 (7/1856)	

*P < 0.05**:** Statistically significant, n: Number of amino acid substitutions, N: Total number of samples genotyped

An evaluation of gene polymorphisms of the *Pfdhfr* revealed that the triple IRN mutant haplotype was the most reported in 18 studies with the minimum prevalence of 4.3% [79] and a maximum prevalence of 100.0% [54, 58]. Only 10 studies reported the quadruple IRNG mutant haplotype involving *Pfdhfr* and *Pfdhps* with the highest prevalence of 95.9% [60]. Moreover, *Pfdhfr*/*Pfdhps* quintuple haplotype IRNGE was identified in 3 studies [58, 60, 79] with a maximum prevalence of 16.7% [58] (Additional file 7).

The *Pfdhfr* IRN and *Pfdhfr*/*Pfdhps* IRNGE haplotypes remained largely unchanged from 66.2% to 67.3 (P = 0.427) and 0.0% to 0.3% (P = 0.623), respectively, between 1998 and 2020. Conversely, a significant decrease in trend from 47.5% to 28.7% was reported for *Pfdhfr-Pfdhps* IRNG under the same period (P < 0.0001) (Table 4).

**Table 4:** Changes in the frequency of *Pfdhfr* and *Pfdhps* haplotypes between 1998 and 2020

Gene	Haplotype	1998–2008 (%, n/N)	2009–2020 (%, n/N)	P-value
*Pfdhfr*	IRN	66.2 (1,821/2,751)	67.3 (1,295/1,924)	P = 0.427
*Pfdhfr-Pfdhps*	IRNG	47.5 (207/436)	28.7 (141/492)	**P < 0.0001**
*Pfdhfr-Pfdhps*	IRNGE	0.0 (0/81)	0.3 (5/1,681)	P = 0.623

*P < 0.05: Statistically significant, n: Number of amino acid substitutions, N: Total number of samples genotyped

### Distribution of antifolate haplotypes across geo-ecological zones of Cameroon

The forest and Sudano-Sahelian zones constitute the major geo-ecological zones of Cameroon. The characteristics of these geo-ecological zones are reflected in the towns of Yaoundé, Mutengene and Garoua. Different studies were conducted in Yaoundé, Mutengene and Garoua from 2004–2006 in order to understand the evolutionary origins of the antifolate haplotypes [46, 86]. The prevalence of *Pfdhfr* CIRN mixed haplotype in the different towns was distributed as follows: Yaounde-70.7%, Mutengene-83.2% and Garoua-21.3%. The *Pfdhps* SGK mixed haplotype was also common in Yaounde-36.3% and Mutengene-66.4% with the least occurrence reported in Garoua-8.3%. The SGK was associated with SP resistance at these three sites. The wild-type alleles (SAK and AAK) were mostly noticeable in Garoua, Yaoundé and Mutengene, respectively. The CIRN haplotype was highly prevalent in the Southern part when compared to the Northern part of Cameroon.

**Fig. 4:**
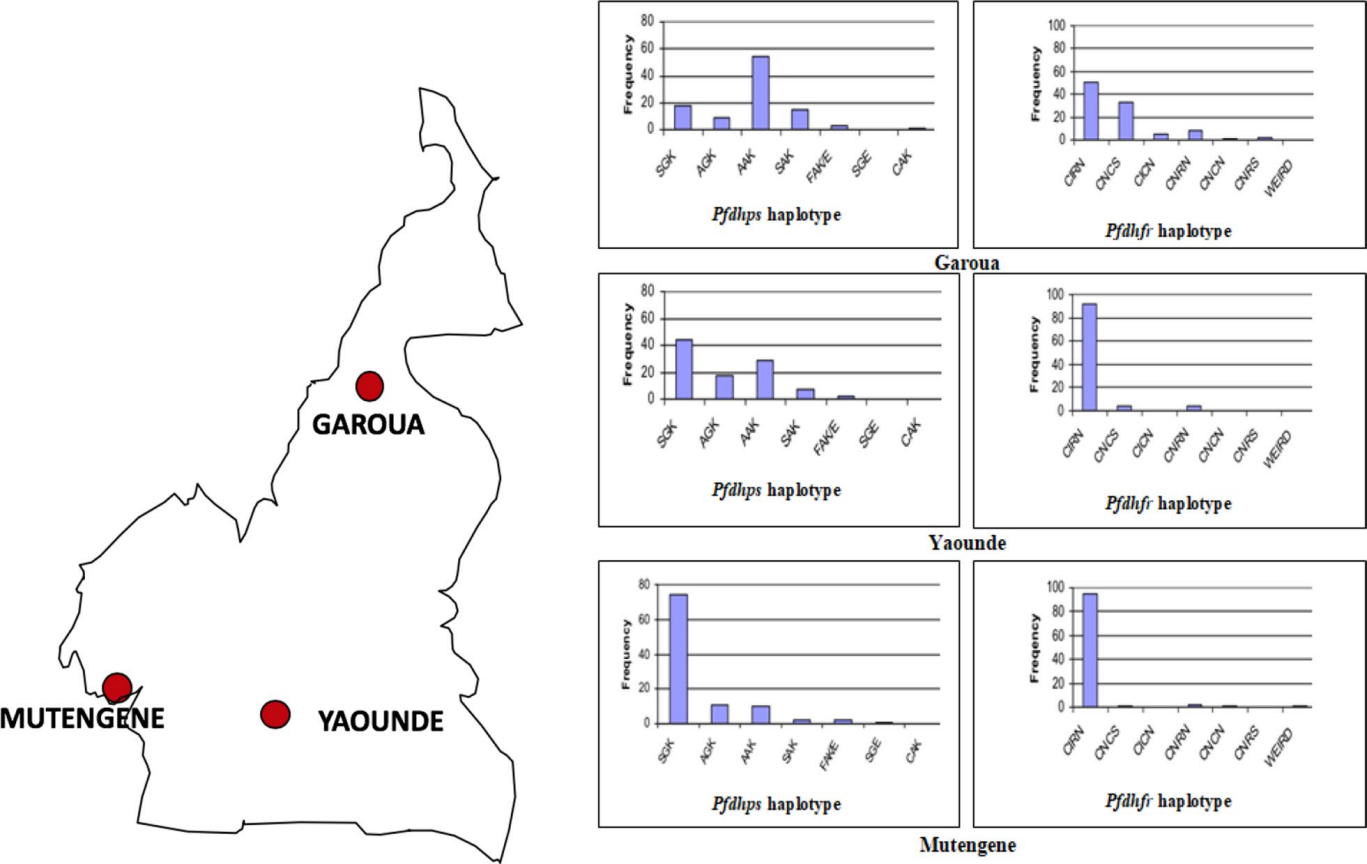
*Pfdhfr* and *Pfdhps* haplotype distribution in three major towns of Cameroon. Efficacy of ACTs (AL and ASAQ) and prevalence of *Pfcrt* 76 T and *Pfmdr1* 86Y mutants over time

Generally, opposing trends were observed in the haplotypes SGK, AGK, AAK, SAK, CIRN, CNCS, CICN, and CNRN in malaria parasites isolated from Garoua, Yaoundé and Mutengene. Data is not available on these unique mixed haplotypes from other regions in Cameroon (Fig. 4).

### Efficacy of ACTs (AL and ASAQ) and prevalence of *Pfcrt* 76 T and *Pfmdr1* 86Y mutants over time

**Fig. 5:**
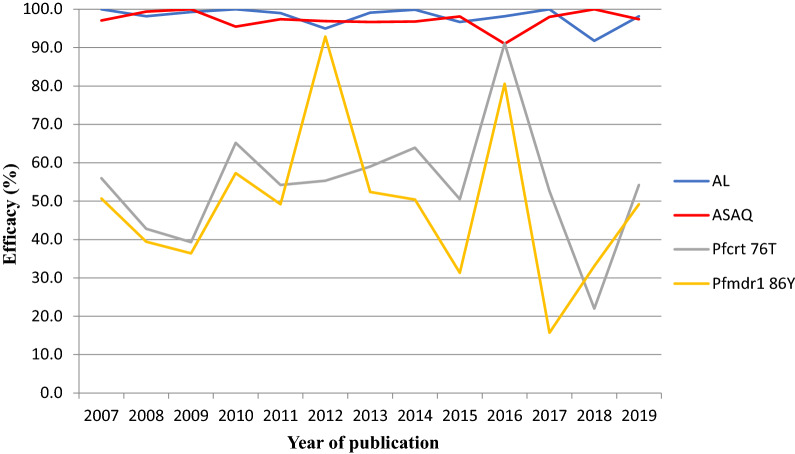
Efficacy of AL/ASAQ and prevalence of *Pfcrt* 76 T and *Pfmdr1* 86Y mutant alleles from 2008 to 2019. AL, Artemether–lumefantrine, ASAQ, Artesunate-amodiaquine, *Pfcrt*, *Plasmodium falciparum* chloroquine resistance transporter gene, *Pfmdr1*, *Plasmodium falciparum* multidrug resistance 1 gene

A total of 13 (3 unpublished and 10 published) studies were used to derive the data on the efficacy (PCR-corrected cure rates) of AL and ASAQ [87–96]. The efficacy rates for AL and ASAQ were above 90.0% and remained relatively constant from 2008–2019. On the contrary, there was a general decline in the *Pfcrt* 76 T and *Pfmdr1* 86Y mutant alleles between 2008 and 2019 which were more pronounced between 2012 and 2016 for *Pfmdr1* and between 2016 and 2019 for *Pfcrt* 76 T. The efficacy of AL showed a positive but non-significant correlation with *Pfcrt* 76 T mutant allele (r = 0.512, P = 0.073) while the efficacy of AL demonstrated a negative non-significant relationship with *Pfmdr1* 86Y mutant allele (r = −0.107, P = 0.728). However, there was a negative significant correlation between the efficacy of ASAQ and prevalence rates of *Pfcrt* 76 T mutant allele (r =  −0.949, P < 0.0001) and *Pfmdr1* 86Y mutant allele (r = −0.657, P = 0.015). Generally, the prevalence of *Pfcrt* 76 T and *Pfmdr1* 86Y mutant alleles were below the efficacy rates of ASAQ and AL (Fig. 5). This showed that increase in mutant alleles corresponded with decrease in efficacy of ACTs and vice versa.

### Trend analysis of proportions of anti-malarial drugs (ASAQ, AL and SP) deployed in Cameroon and prevalence of drug resistance markers from 2006 to 2017

**Fig. 6:**
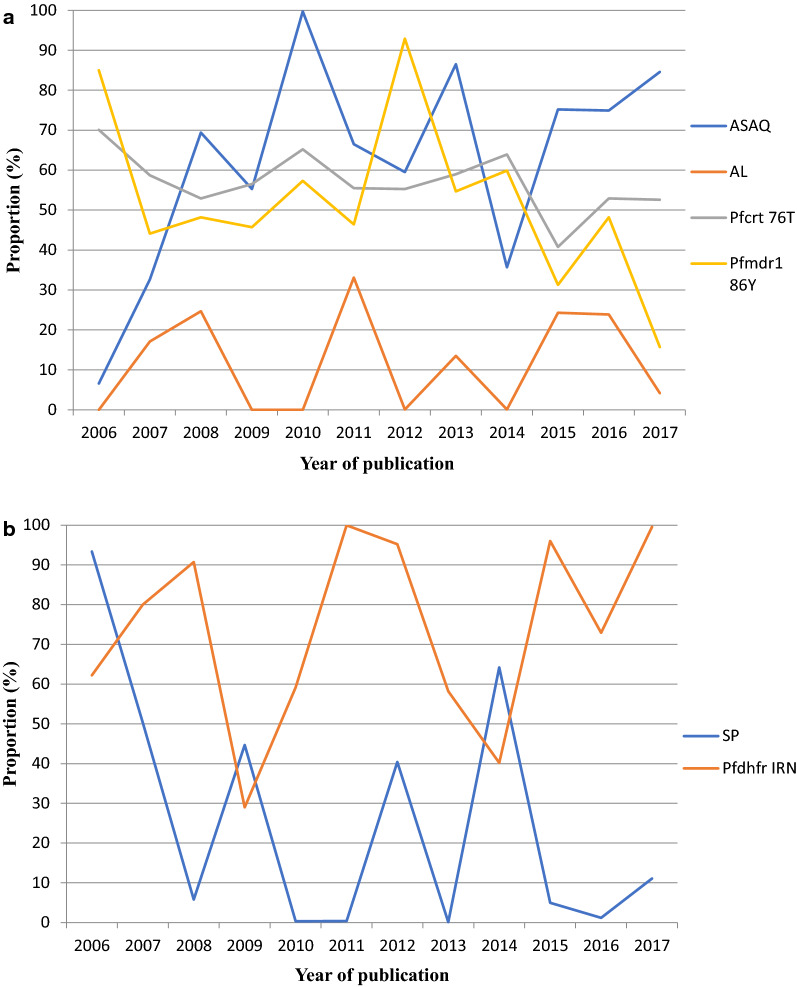
**a** Proportion of ASAQ and AL deployed in Cameroon versus prevalence of *Pfcrt* 76 T and *Pfmdr1* 86Y mutants from 2006 to 2017. ASAQ: Artesunate-amodiaquine, AL, Artemether–lumefantrine; *Pfcrt*, *Plasmodium falciparum* chloroquine resistance transporter gene; *Pfmdr1*, *Plasmodium falciparum*, multidrug resistance 1 gene. **b** Proportion of SP deployed in Cameroon versus prevalence of *Pfdhfr* IRN mutant haplotype from 2006 to 2017. SP, Sulfadoxine–pyrimethamine, *Pfcrt*, *Plasmodium falciparum* chloroquine resistance transporter gene, *Pfmdr1*, *Plasmodium falciparum* multidrug resistance 1 gene

The proportion of ASAQ, AL and SP was based on the observed frequency of each drug deployed to the different health facilities in Cameroon by the NMCP through CENAME and the Regional Funds for Health Promotions. The data was derived from annual reports published by the NMCP. Between 2006 and 2017, there was an increase in the proportion of ASAQ (6.6%-84.6%). Peak distributions for ASAQ were observed in 2010 (99.7%) and 2013 (86.5%) with corresponding prevalence of *Pfcrt* 76 T (65.2%, 59.0%) and *Pfmdr1* 86Y (57.3%, 54.7%) mutants. The proportions of AL distributed from 2006 to 2017 were low when compared with ASAQ. The maximum proportion of AL deployed was 33.1% in 2011 and this corresponded with a prevalence of 55.5% and 46.4% for *Pfcrt* 76 T and *Pfmdr1* 86Y mutants respectively. The Pearson correlation coefficients revealed negative relationships between the ACTs and anti-malarial drug resistance markers [(ASAQ *versus Pfcrt* 76 T, r =  − 0.460, P = 0.133; ASAQ *versus Pfmdr1* 86Y, r =   0.464, P =  − 0.129), (AL *versus Pfcrt* 76 T, r =  − 0.584, P = 0.046; AL *versus Pfmdr1* 86Y, r = − 0.415, P = 0.180) (Fig. 6a).

The proportion of SP deployed to the different Regions of Cameroon dropped from 93.4% in 2006 to 11.1% in 2017 which corresponded with the prevalence of 62.2% and 99.6% of *Pfdhfr* IRN triple mutant haplotype. There was a negative correlation between proportion of SP deployed and prevalence of *Pfdhfr* IRN triple mutant haplotype (r =  − 0.423, P = 0.171).

## Discussion

This systematic review and meta-analysis showed the frequency and geographic distribution of anti-malarial drug resistance markers over a period of three decades in Cameroon. The present study showed that the pooled prevalence of all the amino acid changes from 1998 to 2020 was 35.4%. Subgroup analyses revealed that the aggregated prevalence of *Pfcrt* K76T, *Pfmdr1* N86Y, *Pfdhfr* IRN, and *Pfdhfr-Pfdhps* IRNG were above 40.0% with the exception of *Pfk13*. These analyses highlight the dominance of *Pfcrt* K76T, *Pfmdr1* N86Y, *Pfdhfr* N51I, *Pfdhfr* C59R, *Pfdhfr* S108N, *Pfdhps* A437G and *Pfk13* K189T mutations. The rates are high and further confirm that resistant parasites are still circulating in towns, such as Yaoundé, Garoua, Mutengene, and Buea. This is not surprising considering some of these towns (Yaoundé, Mutengene and Buea) are located within the high malaria transmission stratum and are urban settings with high variability and intensity in the use of anti-malarial drugs with insufficient regulation. It is also around these areas that the first cases of resistance to chloroquine were reported in the 1980s and early 2000 that eventually spread to other Regions [97–100]. The dispersal of drug resistance markers could be due to human and vector population migration within the same Region or between different Regions. The presence of drug resistance markers has been regularly reported in the Southern Regions of Cameroon where malaria transmission is perennial compared to the Northern Regions characterized by intense seasonal transmission.

Previous studies have demonstrated the association of *Pfcrt* 76 T and *Pfmdr1* 86Y mutant alleles with chloroquine and amodiaquine resistance in vivo among uncomplicated *falciparum* malaria patients in different transmission settings [8, 13]. These two drugs, chloroquine and amodiaquine were banned and withdrawn from the market since 2002 and 2004 respectively for the treatment of uncomplicated *falciparum* malaria in Cameroon. However, amodiaquine (AQ) continues to be used as a partner drug in the artesunate-amodiaquine (ASAQ) and sulfadoxine–pyrimethamine–amodiaquine (SPAQ) combinations. In 2004, ASAQ combination replaced AQ and SP for the treatment of uncomplicated *falciparum* malaria in the Southern Regions while SPAQ was introduced in 2016 as chemoprophylaxis in the context of seasonal malaria chemoprevention among children 3–59 months in the North and Far north Regions of Cameroon. The most common quintuple haplotypes identified in *Pfcrt* gene were CVMNK and CVIET. This concords with previously published studies in other regions [101, 102]. It is important to note that one study reported the presence of *Pfcrt* SVMNT haplotype with a prevalence of 4.4% [59], which is lower than the 19.0% [15] and 56.9% [14] reported in the Korogwe District, Tanzania and Luanda, Angola, respectively.

Only two studies reported the triple *Pfmdr1* NFD haplotype [45, 57] while the triple *Pfmdr1* YYY haplotype was not documented. A number of studies carried out in malaria endemic areas have demonstrated an opposing effect in the selection of YYY for ASAQ and NFD for AL [22, 103]. This is advantageous to Cameroon since ASAQ and AL are used as multiple first-line treatments (MFTs) that can possibly slow down the emergence of drug resistance [104].

Trend analysis showed that *Pfcrt* 76 T, *Pfcrt* CVMNK quintuple wild type haplotype, and *Pfmdr1* 86Y mutant parasites declined from 1998–2020. This is in agreement with previous studies carried out in other malaria endemic zones confirming the re-emergence of chloroquine sensitive parasites [101, 102, 105]. However, there should be caution in the future use of chloroquine in the treatment of uncomplicated *P*. *falciparum* malaria because this may lead to reintroduction of resistant parasites population.

In Cameroon, sulfadoxine–pyrimethamine (SP) is still being deployed as intermittent preventive treatment for malaria in pregnancy (IPTp) with estimated coverage of about 32% in 2018 [106]. The antifolates are also used in combination with amodiaquine for seasonal malaria chemoprevention. The presence of mutations in *Pfdhfr* and *Pfdhps* genes conferring resistance to SP does not seem to threaten the continuous use of this drug in the future especially as there is need to scale-up deployment to pregnant women and young children as intermittent preventive treatment (IPTp and IPTi). This may also be applicable for children receiving SPAQ in the context of seasonal malaria chemoprevention in the Sahel regions of Northern Cameroon. The triple *Pfdhfr* IRN and quadruple *Pfdhfr*/*Pfdhps* IRNG mutant haplotypes were the most prevalent while quintuple *Pfdhfr*/*Pfdhps* IRNGE mutant haplotype was the least prevalent. There has been a gradual decline over the years in the prevalence of single antifolate gene polymorphisms associated with SP resistance in Cameroon with the exception of *Pfdhps* A437G and K540E. However, the rates of prevalence recorded are less than the 90% benchmark recommended by the WHO to ban the continuous use of SP. These findings corroborate with the high prevalence of *Pfdhfr* IRN and *Pfdhfr*/*Pfdhps* IRNG recorded in Bata District and Bioko Island, Equatorial Guinea [107, 108]. There has been a gradual increase in the prevalence of the quintuple *Pfdhfr*/*Pfdhps* IRNGE mutant haplotype over the years, ascertaining the sudden emergence of the haplotype in Central Africa [107]. Other underreported *Pfdhp*s haplotypes included SGK, AGK, SGE, AAK, and SAK. These haplotypes were extensively studied in isolates from different African countries including Cameroon by Pearce and colleagues, where they sought to investigate the evolutionary origin of the mutations flanking the *Pfdhps* gene [86]. The authors observed that the haplotypes in the Cameroonian samples were unique when compared to those from Central, South-eastern and West African sites [86]. The malaria parasite resistance to SP seems to be driving in opposite directions with high resistance recorded in the Southern Regions when compared to the Northern Regions. The location of these sites in different malaria transmission settings may be accountable for the variations observed.

A new mutation, I431V, recently identified in the *Pfdhps* gene has been reported in Yaoundé [60] and Mutengene [57] with prevalence rates of 16.3% and 18.3%, respectively. These rates are lower than that reported in Enugu Nigeria (46.0%) in 2016 [109], suggesting the possibility of different mutant haplotypes associated with SP treatment failure in Central/West Africa. This is unlike previous observations in East Africa where the quintuple *Pfdhfr*/*Pfdhps* IRNGE mutant haplotype is strongly associated is SP resistance [110].

There was the absence of key gene polymorphisms located in the *Pfk13* propeller region, F446I, R539T, I543T, P574L and C580Y previously documented in the Greater Mekong sub-region which are associated with delayed parasite clearance following drug administration. Moreover, a negative or positive relationship was observed between the rate of efficacy of ASAQ/AL and the prevalence of key mutants (*Pfcrt* K76T and *Pfmdr1* N86Y) that select for the partner drugs in ACT. These observations confirm the findings that AL and ASAQ exert opposing selective effects on single-nucleotide polymorphisms in *Pfcrt* and *Pfmdr1* [21]. However, ASAQ and AL are still efficacious with rates of efficacy above the WHO minimum cut-off of 90%.

It has been shown that some individuals infected with drug resistant parasites are still able to clear the parasites when administered with non-ACT and ACT [111, 112]. This may be due to immune competence of such individuals. Semi-immune individuals have an enhanced ability to clear faster than non-immune people. In addition, age has also been identified as a contributory factor with children less than 5 years clearing parasites slower when compared to children greater than five years [113]. Even though immunity due to malaria infection is short-lived, certain cytokines and their receptors have been shown to be highly implicated in this process [111, 112].

Furthermore, there was a negative correlation between the proportions of anti-malarial drugs (ASAQ, AL and SP) deployed to the different public and private health establishments in Cameroon and anti-malarial drug resistance markers (*Pfcrt* 76T, *Pfmdr1* 86Y and *Pfdhfr* IRN). The proportion of drugs deployed may be used as a proxy for drug uptake. The decline in proportion of drugs deployed may be contributing to less drug pressure to circulating parasites. Increase in parasite fitness as a result of less drug pressure could be responsible for the decline in the prevalence of certain gene mutations associated with anti-malarial drug resistance. The two drugs, ASAQ and SP are still being subsidized by the Cameroon government. ASAQ is highly recommended for the treatment of uncomplicated *falciparum* malaria in children less than 5 years while SP used as a preventive treatment for malaria in pregnancy. The major challenge in the fight against drug resistance in Cameroon is the inability to effectively implement the legislation on the homologation and importation of unauthorized anti-malarial therapies and insufficient pharmacovigilance. In addition, there are still issues with substandard drugs and auto-medication.

### Strengths and limitations of the study

The major strength of the present review is that it has presented a picture of the prevalence and distribution of key anti-malarial drug resistance markers in Cameroon with a total of 48 studies included. The data derived from this study showed that there is little or absence of the *Pfmdr1* and *Pfk13* polymorphisms that select for ACT, especially ASAQ and AL. These 2 drugs are used concurrently for the management of uncomplicated *Plasmodium falciparum* malaria in Cameroon.

However, despite the strengths of the study, it is not without limitations. Firstly, some studies enrolled a fewer number of participants which may not give a true representation of resistant parasite population circulating in the Cameroon. Secondly, the high heterogeneity across studies may affect the interpretation of the findings. Thirdly, some of anti-malarial drug resistance markers have been understudied in the Northern Regions of the country that border countries such as Nigeria with a high burden of malaria. Furthermore, most of the studies were conducted in symptomatic individuals and there is little or no information on the prevalence of anti-malarial drug resistance markers in asymptomatic carriers of the parasite. Asymptomatic individuals have been shown to be reservoirs for malaria parasite transmission. In addition, earlier studies mostly used nPCR-RFLP for the detection of drug resistance markers and, therefore, were not capable of identifying novel SNPs. Finally, the association between specific *P*. *falciparum* gene polymorphisms and treatment failures with ACT could not be investigated because of the non-availability of data.

## Conclusions

This review reported a decline in the prevalence of single *Plasmodium falciparum* gene mutations (*Pfcrt* K76T, *Pfmdr1* N86Y, *Pfdhfr* N51I, *Pfdhfr* C59R, *Pfdhfr* S108N) conferring resistance to 4-aminoquinolines, amino alcohols and pyrimethamine for a period over two decades pre and post adoption of ACT in Cameroon. The *Pfcrt* K76T and *Pfmdr1* N86Y mutations still persist at moderate frequencies despite the withdrawal of chloroquine. Conversely, parasite resistance markers (*Pfdhps* A437G and *Pfdhps* K540E) linked to the sulpha drugs increased during the same study period. Resistance to artemisinins measured by the presence of SNPs in the *Pfk13* gene does not seem to be a major problem in Cameroon. However, it is a wake-up call for policy makers to design and implement strategies for the regular monitoring of delayed parasite clearance after administration of artemisinin-based combination therapy. This will permit the early identification of factors driving the emergence and spread of anti-malarial drug resistance in Cameroon.

## Supplementary Information

**Additional file 1.** Summary of the studies included in the systematic review and meta-analysis on anti-malarial drug resistance markers in Cameroon.

**Additional file 2.** Methodological quality assessment of interventional studies.

**Additional file 3.** Methodological quality assessment of cohort studies.

**Additional file 4.** Methodological quality assessment of cross-sectional studies.

**Additional file 5.** Methodological quality assessment of case reports.

**Additional file 6.** Assessment of publication bias using funnel plot and Egger’s regression test.

**Additional file 7.** Haplotype analyses of anti-malarial drug resistance mutant allele frequencies reported in Cameroon.

## Abbreviations

ACT: Artemisinin-based combination therapy
AL: Artemether–lumefantrine
ASAQ: Artesunate-amodiaquine
NMCP: National Malaria Control Programme
PCR: Polymerase Chain Reaction
*Pfcrt*: *Plasmodium falciparum* Chloroquine resistance transporter
*Pfmdr1*: *Plasmodium falciparum *Multi-drug resistance 1
*Pfdhfr*: *Plasmodium falciparum *Dihydrofolate reductase
*Pfdhps*: *Plasmodium falciparum *Dihydropteroate synthase
*Pfcytb*: *Plasmodium falciparum *Cytochrome b
*Pfatp6*: *Plasmodium falciparum *Atpase 6
*Pfk13*: *Plasmodium falciparum* Kelch 13
PRISMA: Preferred Reporting Items for Systematic Reviews and Meta-analyses
PRISMA-P: Preferred Reporting Items for Systematic Reviews and Meta-analyses protocol
r: Correlation coefficient
SP: Sulfadoxine–pyrimethamine
SPAQ: Sulfadoxine–pyrimethamine–amodiaquine
WHO: World Health Organization
