# Supplementary material for: Drug resistance markers within an evolving efficacy of anti-malarial drugs in Cameroon: a systematic review and meta-analysis (1998–2020)
Source: Malar J. 2021 Jan 9;20:32. doi: 10.1186/s12936-020-03543-8 (PMC7796563; doi:10.1186/s12936-020-03543-8)
Supplement: Supplementary file 6 — Additional file 6. Assessment of publication bias using funnel plot and Egger’s regression test. [file 12936_2020_3543_MOESM6_ESM.docx]

**Assessment of Publication Bias using Funnel Plot and Egger’s Regression Test**

1. **The pooled prevalence of all amino acid changes in anti**-**malarial drug resistance genes**


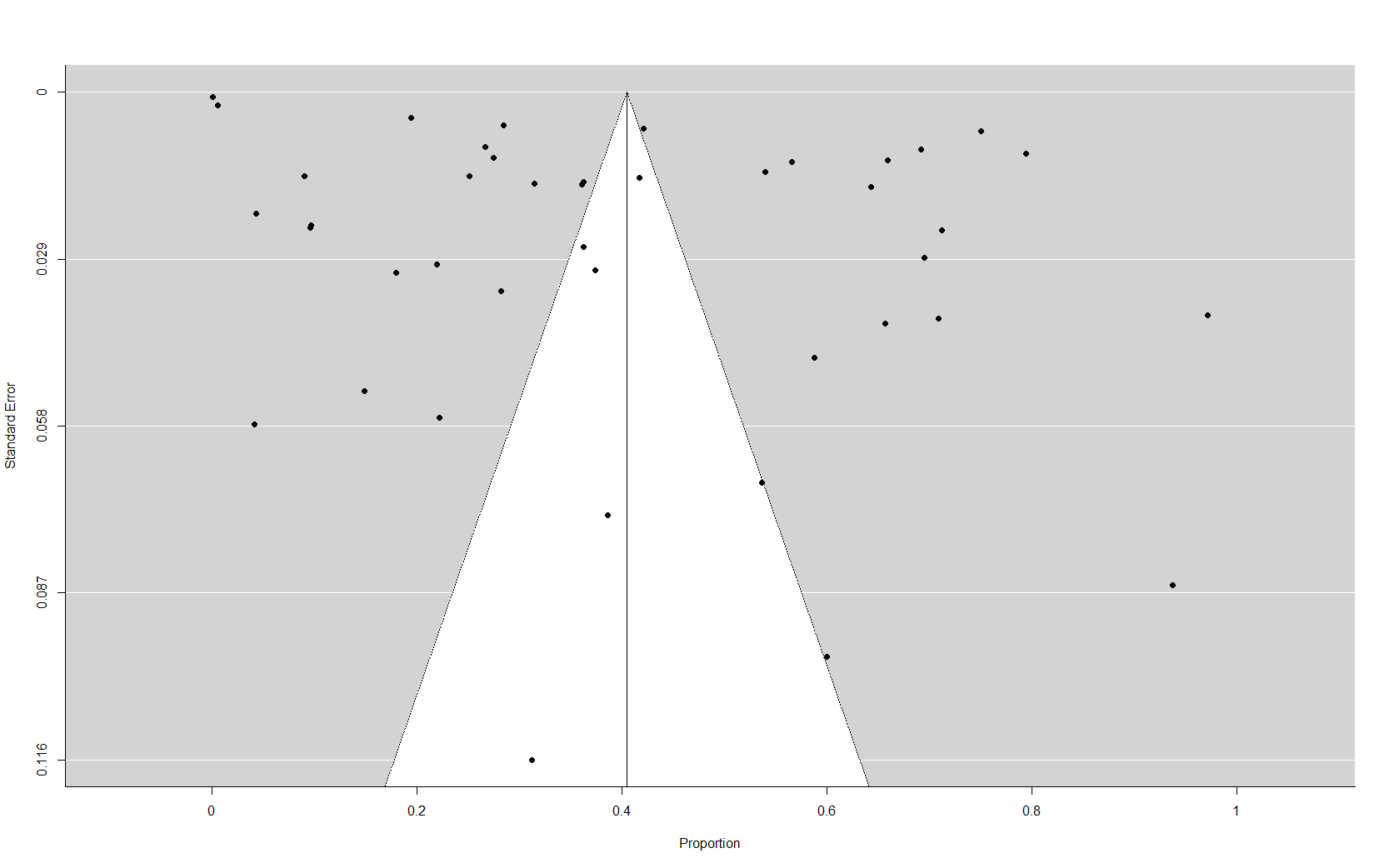


**Figure 1: Funnel plot for pooled prevalence of all amino acid changes in anti**-**malarial drug resistance genes**

Egger’s regression test for funnel plot asymmetry: t=5.6113, df=40, P<0.0001

1. **The pooled prevalence of *Pfcrt* K76T mutations**

**Figure 2: Funnel plot for pooled prevalence of** ***Pfcrt* K76T mutations**

Egger’s regression test for funnel plot asymmetry: t=**-**0.8754, df =19, P=0.3923

1. **The pooled prevalence of *Pfcrt* CVIET mutations**

**Figure 3: Funnel plot for pooled prevalence of** ***Pfcrt* CVIET mutations**

Egger’s regression test for funnel plot asymmetry: t=1.1479, df=7, P= 0.2887

1. **Pool prevalence of *Pfmdr1* N86Y mutations**

**Figure 4: Funnel plot for pool prevalence of** ***Pfmdr1* N86Y mutations**

Egger’s regression test for funnel plot asymmetry: t=-1.3608, df=14, P = 0.1951

1. **Aggregate prevalence of** ***Pfdhfr* IRN haplotype mutations**

**Figure 5: Funnel plot for pooled prevalence of** ***Pfdhfr* IRN mutations**

Egger’s regression test for funnel plot asymmetry: t=**-**3.5659, df=24, P= 0.0016

1. **Pooled prevalence of** ***Pfdhfr*-*Pfdhps* IRNG haplotype mutations**


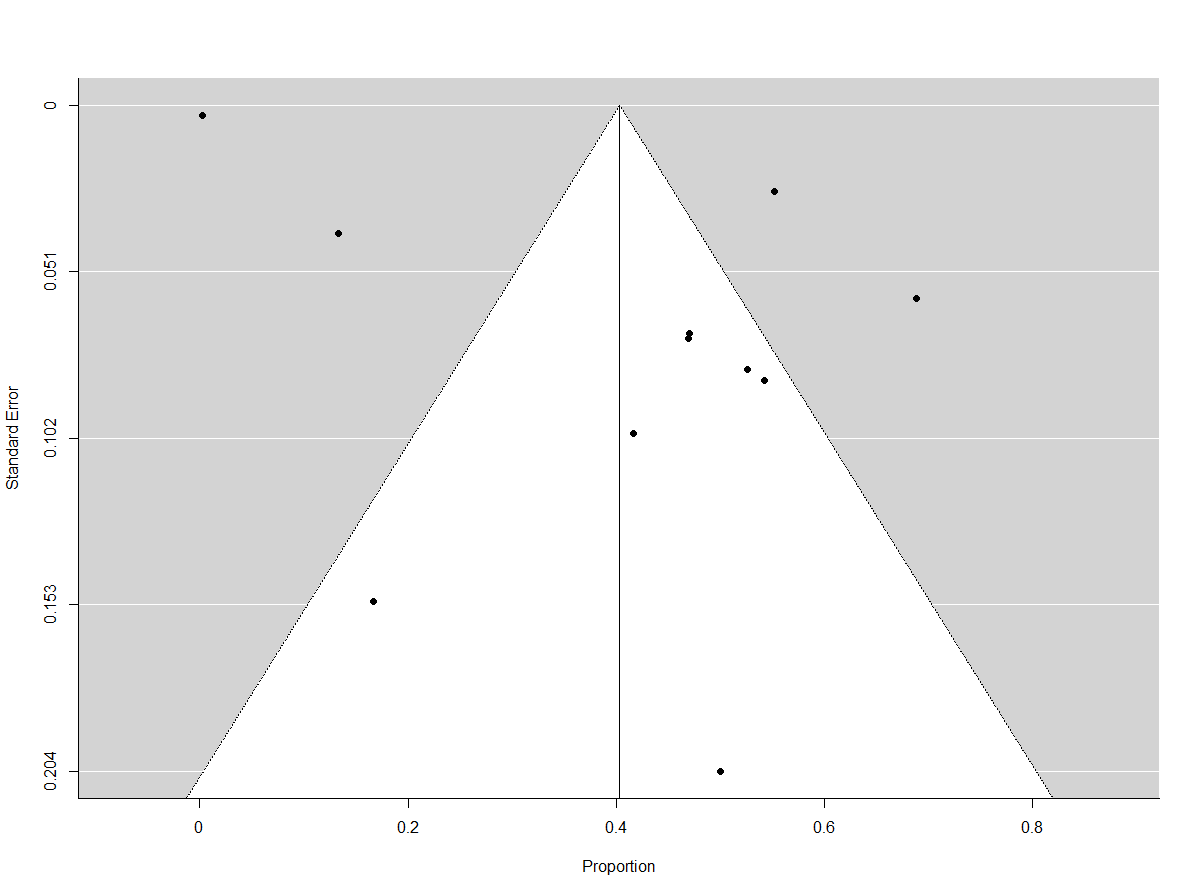


**Figure 6: Forest plot pooled prevalence of** ***Pfdhfr*-*Pfdhps* IRNG haplotype mutations**

Egger’s regression test for funnel plot asymmetry: t = 3.6156, df = 9, P = 0.0056

1. **Pooled prevalence of** ***Pfk13* mutations**


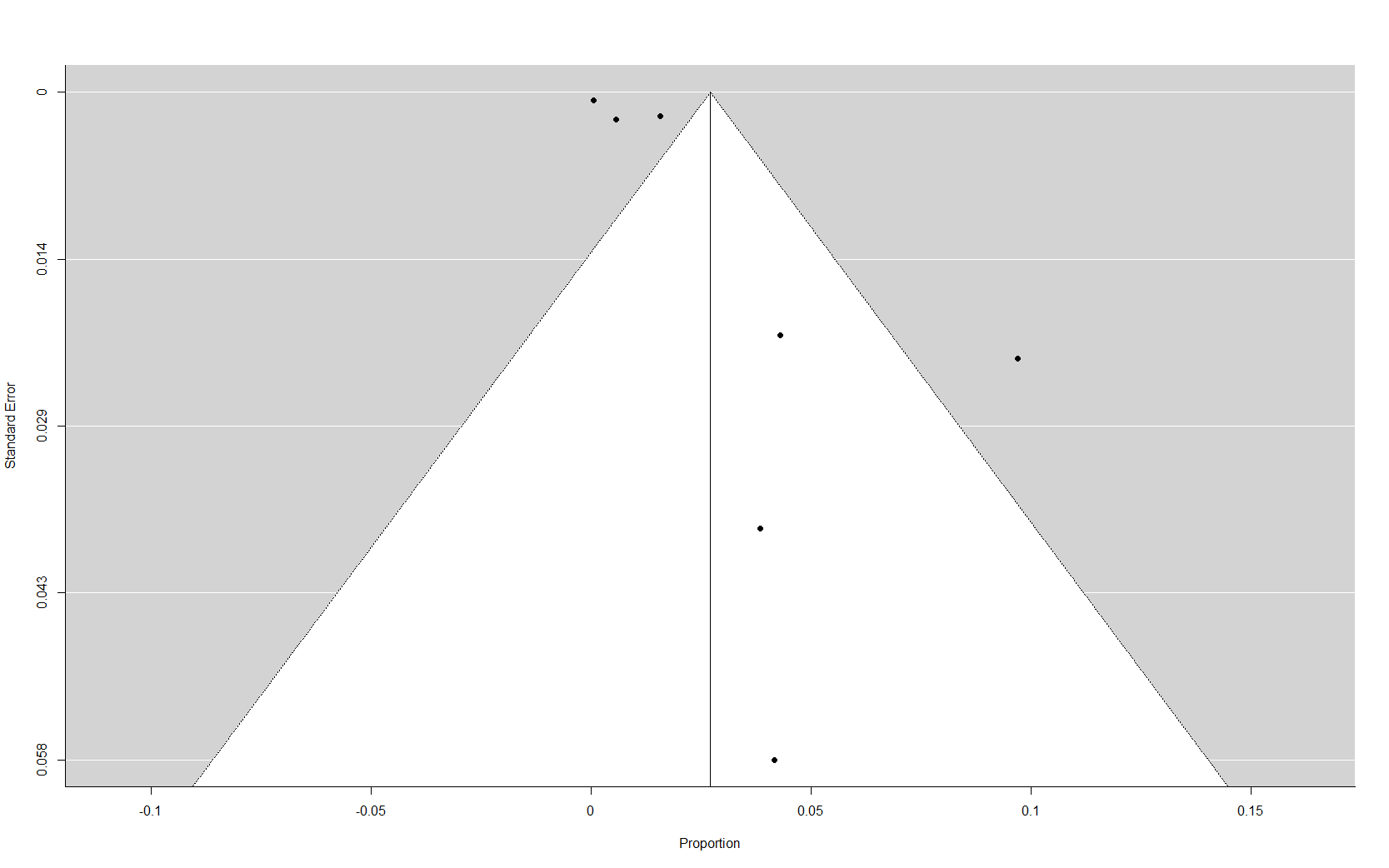


**Figure 7: Funnel plot for pool prevalence of** ***Pfk13* mutations**

Egger’s regression test for funnel plot asymmetry: t = 2.1973, df = 5, P=0.0794
