# Supplementary material for: Drug resistance markers within an evolving efficacy of anti-malarial drugs in Cameroon: a systematic review and meta-analysis (1998–2020)
Source: Malar J. 2021 Jan 9;20:32. doi: 10.1186/s12936-020-03543-8 (PMC7796563; doi:10.1186/s12936-020-03543-8)
Supplement: Supplementary file 7 — Additional file 7. Haplotype analyses of anti-malarial drug resistance mutant allele frequencies reported in Cameroon. [file 12936_2020_3543_MOESM7_ESM.docx]

**Haplotype frequencies of *Pfcrt* wild type and mutant alleles reported in Cameroon from 1998**-**2019**

| **Author and year of publication** | **Site of study** | **Year of study** | **Sample size** | ***Pfcrt* mutation (%)** | | | | | **CVMNK (%)** | **CVIET (%)** | **SVMNT (%)** |
| --- | --- | --- | --- | --- | --- | --- | --- | --- | --- | --- | --- |
|  |  |  |  | **72S** | **73K** | **74I** | **75E** | **76T** |  |  |  |
| Basco and Ringwald, 2001 | Yaounde | 1994**-**1998 | 67 | ND | ND | ND | ND | 88.1 | ND | ND | ND |
| Basco, 2002 | Yaounde | 2000**-**2001 | 95 | 93.7 | 93.7 | 93.7 | 93.7 | 93.7 | 6.3 | 93.7 | ND |
| Basco, 2002 | Yaounde | 2000**-**2001 | 118 | 57.6 | ND | ND | ND | ND | ND | ND | ND |
| Basco *et al*., 2002 | Maroua, Ndop, Bafoussam, and Hevecam | 2000**-**2001 | 61 | 0.0 | 0.0 | 0.0 | 0.0 | 90.2 | 9.8 | 0.0 | ND |
| de Monbrison *et al*., 2003 | Travellers, Cameroon, Indonesia | ND | 12 | ND | ND | 92.3 | 92.3 | 92.3 | ND | ND | ND |
| Severini *et al*., 2005 | Cameroon and other African countries | 1998**-**2003 | 5 | 0.0 | 80.0 | 80.0 | 80.0 | 80.0 | 20.0 | 0.0 | 0.0 |
| Mbacham *et al*., 2010 | Mutengene | 2004**-**2006 | 256 | ND | ND | ND | ND | 87.1 | ND | ND | ND |
| Mbacham *et al*., 2010 | Yaounde | 2004**-**2006 | 259 | ND | ND | ND | ND | 76.8 | ND | ND | ND |
| Mbacham *et al*., 2010 | Garoua | 2004**-**2006 | 240 | ND | ND | ND | ND | 31.7 | ND | ND | ND |
| Menard *et al*.. 2012 | Yaounde | 2005**-**2009 | 216 | 0.0 | ND | ND | ND | 81.3 | ND | ND | ND |
| Menard *et al*., 2012 | Mfou | 2005**-**2009 | 231 | 0.0 | ND | ND | ND | 84.7 | ND | ND | ND |
| Gharbi *et al*., 2013 | Senegal, Mali, Ivory Coast and Cameroon | 2001**-**2011 | 719 | ND | ND | ND | ND | 59.0 | ND | ND | ND |
| Mbenda and Das, 2014 | Ebolowa, Douala, Yaounde, Bertoua and Kye-ossi | ND | 180 | 4.4 | 0.0 | 55.6 | 56.7 | 63.9 | 55.6 | 36.1 | 4.4 |
| Ndam *et al*., 2017 | Bertoua | 2003 | 164 | 18.9 | 72.0 | 72.0 | 72.0 | 72.0 | 28.0 | 53.0 | ND |
| Ndam *et al*., 2017 | Messok | 2012 | 95 | 21.1 | 46.3 | 46.3 | 46.3 | 46.3 | 53.7 | 25.3 | ND |
| Lu *et al*., 2017 | Cameroon and others | 2011**-**2014 | 11 | NA | NA | 36.4 | 36.4 | 36.4 | 63.6 | 36.4 | NA |
| Apinjoh *et al*., 2017 | Mutengene, Ombe,Tiko, Mile 14, Mile 15, Mile 16, Buea Town and Tole, Cameroon | 2013**-**2014 | 254 | 0.0 | 0.0 | 55.4 | 54.9 | 55.7 | 25.3 | 74.7 | 0.0 |
| Moyeh *et al*., 2018 | Mutengene | 2009**-**2013 | 260 | NA | NA | NA | NA | 32.3 | NA | NA | NA |
| Achungu *et al*., 2018 | Bamenda | NA | 191 | NA | NA | NA | NA | 22.0 | 78.0 | NA | NA |
| Yao *et al*., 2018 | Cameron, other African and Southeast Asian countries | 2011**-**2016 | 7 | 0.0 | 0.0 | 14.3 | 14.3 | 14.3 | 85.7 | 0.0 | 0.0 |
| Zhang *et al*., 2018 | Cameroon and others African countries | 2012**-**2016 | 26 | NA | 3.8 | NA | 19.2 | NA | NA | NA | NA |

ND: No data available

**Haplotype frequencies of *Pfmdr1* wild type *and* mutant alleles reported in Cameroon from 1998**-**2019**

| **Authors and year of publication** | **Site of study** | **Year of study** | **Sample size** | ***Pfmdr1* mutation (%)** | | | **YFY (%)** | **YYY (%)** | **NFD (%)** | **Copy number variation** | |
| --- | --- | --- | --- | --- | --- | --- | --- | --- | --- | --- | --- |
|  |  |  |  | **86Y** | **184F** | **1246Y** |  |  |  |  |  |
|  |  |  |  |  |  |  |  |  |  | **≤1** | **>1** |
| Basco and Ringwald, 1998 | Yaounde | 1994**-**1995 | 129 | 90.7 | ND | ND | ND | ND | ND | ND | ND |
| Basco and Ringwald, 1999 | Yaounde | 1994**-**1995 | 55 | 76.4 | ND | ND | ND | ND | ND | ND | ND |
| Basco and Ringwald, 2001 | Yaounde | 1994**-**1998 | 67 | 53.7 | ND | ND | ND | ND | ND | ND | ND |
| Basco and Ringwald, 2002 | Yaounde | 1997**-**2000 | 64 | 89.1 | 92.2 | 0.0 | 0.0 | 0.0 | ND | ND | ND |
| de Monbrison *et al*., 2003 | Travellers, Cameroon, Indonesia | ND | 15 | 100.0 | 100.0 | 6.7 | ND | ND | ND | ND | ND |
| Mbacham *et al*., 2010 | Mutengene, | 2004**-**2006 | 256 | 73.8 | ND | ND | ND | ND | ND | ND | ND |
| Mbacham *et al*., 2010 | Yaounde | 2004**-**2006 | 259 | 76.1 | ND | ND | ND | ND | ND | ND | ND |
| Mbacham *et al*., 2010 | Garoua | 2004**-**2006 | 240 | 22.1 | ND | ND | ND | ND | ND | ND | ND |
| Witkowski *et* *al*., 2010 | Ivory Coast, Burkina Faso, Senegal, Guinea Conakry, Mali, Benin, Togo, Mauritania, Nigeria, Ghana, Liberia, Cameroon, Central Africa Republic, Gabon, Congo and Chad | 2005**-**2009 | 17 | ND | ND | ND | ND | ND | ND | 100.0 | 0.0 |
| Menard *et al*., 2012 | Yaounde | 2005**-**2009 | 216 | 90.0 | ND | ND | ND | ND | ND | 100.0 | 0.0 |
| Menard *et al*., 2012 | Mfou | 2005**-**2009 | 231 | 95.7 | ND | ND | ND | ND | ND | ND | ND |
| Gharbi *et al*., 2013 | Senegal, Mali, Ivory Coast and Cameroon | 1996**-**2011 | 719 | ND | ND | 100.0 | ND | ND | ND | ND | ND |
| Nji *et al*., 2015 | Garoua and Mutengene | 2009**-**2013 | 16 | 31.3 | ND | ND | ND | ND | ND | ND | ND |
| Apinjoh *et al*., 2017 | Mutengene, Ombe,Tiko, Mile 14, Mile 15, Mile 16, Buea Town and Tole, Cameroon | 2013**-**2014 | 254 | 13.4 | 72.2 | 0.0 | 0.0 | 0.0 | 72.2 | ND | ND |
| Ndum *et al*., 2017 | Garoua, Maroua and Yagoua | 2014 | 150 | 18.0 | ND | ND | ND | ND | ND | ND | ND |
| Moyeh *et al*., 2018 | Mutengene | 2009**-**2013 | 260 | 55.8 | 44.0 | 100.0 | ND | ND | 25.2 | ND | ND |
| Yao *et al*., 2018 | Cameron, other African and Southeast Asian countries | 2011**-**2016 | 6 | 16.7 | 50.0 | ND | ND | ND | ND | ND | ND |
| Zhang *et al*., 2018 | Cameroon and others African countries | 2012**-**2016 | 26 | 26.9 | ND | ND | ND | ND | ND | ND | ND |

ND: No data available

**Haplotype frequencies of *Pfdhfr* and *Pfdhps* mutant alleles reported in Cameroon from 1998**-**2019**

| **Author and year of publication** | **Site of study** | **Year of study** | **Sample size** | ***Pfdhfr* mutation (%)** | | | **IRN haplotype (%)** | ***Pfdhps* mutation (%)** | | **IRNG (%)** | **IRNGE (%)** |
| --- | --- | --- | --- | --- | --- | --- | --- | --- | --- | --- | --- |
|  |  |  |  | **51I** | **59R** | **108N** |  | **437G** | **540E** |  |  |
| Basco *et al*., 1998 | Yaounde | ND | 6 | 50.0 | 80.0 | 80.0 | 50.0 | 16.7 | 0.0 | 16.7 | 0.0 |
| Basco and Ringwald, 1998 | Yaounde | 1994**-**1995 | 127 | ND | ND | 48.0 | ND | ND | ND | ND | ND |
| Basco and Ringwald, 1999 | Yaounde | 1997**-**1998 | 85 | ND | ND | 58.8 | ND | ND | ND | ND | ND |
| Basco *et al*., 2000 | Yaounde | ND | 75 | 52.0 | 64.0 | 74.7 | 50.7 | 37.5 | 0.0 | 13.3 | 0.0 |
| Basco *et al*., 2000 | Yaounde | ND | 34 | 41.2 | 47.1 | 52.9 | 41.2 | ND | ND | ND | ND |
| Basco, 2002 | Yaounde | 2000**-**2001 | 118 | 16.1 | 17.8 | 71.2 | 16.1 | ND | ND | ND | ND |
| Basco *et al*., 2002 | Yaounde, Eseka, Bertoua, Douala | 1999 | 175 | 59.4 | 72.0 | 77.7 | 58.3 | ND | ND | ND | ND |
| Basco, 2003 | Yaounde | 2000**-**2001 | 139 | 82.0 | 74.1 | 92.8 | 74.1 | ND | ND | ND | ND |
| Tahar and Basco, 2006 | Yaounde, Eseka, Bertoua, Douala, Ndop, Bafoussam, Maroua, Mengang, Hevecam, Sangmelima, Djoum, Garoua, Ngaoundere, Manjo | 1999**-**2003 | 1368 | 70.6 | 75.5 | 79.1 | 62.2 | ND | ND | ND | ND |
| Tahar and Basco, 2007 | Yaounde, Djoum, Manjo, Bertoua and Garoua Cameroon | 1999**-**2003 | 355 | 81.1 | 84.8 | 87.3 | 74.1 | 45.4 | 0.0 | 55.2 | ND |
| Tahar and Basco, 2007 | Yaounde | 2004**-**2005 | 194 | 92.8 | 95.9 | 96.4 | 90.7 | ND | ND | ND | ND |
| McCollum *et al*., 2008 | Yaounde | 2001**-**2005 | 287 | 88.9 | 92.7 | 95.1 | 87.8 | 65.1 | ND | ND | ND |
| Mbacham *et al*., 2009 | Fontem | 2002 | 100 | 14.0 | 14.0 | 14.0 | 14.0 | 39.8 | 0.0 | ND | 0.0 |
| Mbacham *et al*., 2009 | Dschang | 2002 | 93 | 4.3 | 4.3 | 4.3 | 4.3 | 22.2 | 0.0 | ND | 0.0 |
| Mbacham *et al*., 2009 | Limbe | 2003 | 138 | 44.2 | 44.2 | 44.2 | 44.2 | 59.9 | 1.4 | ND | 1.4 |
| Mbacham *et al*., 2009 | Nkambe | 2003 | 103 | 45.6 | 45.6 | 45.6 | 45.6 | 57.6 | 0.0 | ND | 0.0 |
| Mbacham *et al*., 2010 | Mutengene | 2004**-**2006 | 256 | 83.2 | 83.2 | 83.2 | 83.2 | 66.4 | 0.0 | ND | 0.0 |
| Mbacham *et al*., 2010 | Yaounde | 2004**-**2006 | 259 | 70.7 | 70.7 | 70.7 | 70.7 | 36.3 | 0.0 | ND | 0.0 |
| Mbacham *et al*., 2010 | Garoua | 2004**-**2006 | 240 | 21.3 | 21.3 | 21.3 | 21.3 | 8.3 | 0.0 | ND | 0.0 |
| Menemendengue *et al*., 2011 | Yaounde | 2005 | 61 | 100.0 | 100.0 | 100.0 | 100.0 | 68.9 | 0.0 | 68.9 | 0.0 |
| Ngwafor *et al*., 2015 | Garoua, Maroua and Yagoua | 2014 | 7 | ND | 100.0 | ND | ND | ND | ND | ND | ND |
| Chauvin *et al*., 2015 | Yaounde | 2010**-**2011 | 51 | 96.1 | 98.0 | 98.0 | 94.1 | 76.5 | 0.0 | 47.1 | 0.0 |
| Chauvin  *et al*., 2015 | Yaounde | 2010**-**2011 | 49 | 100.0 | 98.0 | 100.0 | 98.0 | 95.9 | 4.1 | 46.9 | 4.1 |
| Apinjoh *et al*., 2017 | Mutengene, Ombe,Tiko, Mile 14, Mile 15, Mile 16, Buea Town and Tole, | 2013**-**2014 | 226 | 99.6 | 99.6 | 100.0 | 99.6 | 0.0 | 0.0 | 0.0 | 0.0 |
| Moyeh *et al*., 2018 | Mutengene | 2009**-**2013 | 243 | 98.8 | 100.0 | 100.0 | 98.8 | 90.0 | 0.0 | ND | ND |
| Acho *et al*., 2008 | Yaounde | 2008**-**2012 | 38 | 100.0 | 100.0 | 92.1 | 92.1 | 55.3 | 0.0 | 52.6 | 0.0 |
| Acho *et al*., 2018 | Yaounde | 2018 | 35 | 100.0 | 100.0 | 100.0 | 100.0 | 65.5 | 0.0 | 54.3 | 0.0 |
| Xu *et al*., 2019 | Nigeria, Ghana, Guinea, Equatorial Guinea, Cameroon, Angola, Mozambique | 2013**-**2016 | 6 | 100.0 | 100.0 | 100.0 | 100.0 | 83.3 | 16.7 | 50.0 | 16.7 |
| Zhao *et al*., 2019 | Liberia, Sierra Leone, Mali, Ghana, Congo, Cameroon, Democratic Republic of Congo, Central African Republic | 2016**-**2018 | 24 | 91.7 | 91.7 | 91.7 | 91.7 | 56.0 | 0.0 | 42.1 | 0.0 |

ND: No data available
